# TAFRO syndrome is associated with anti-SSA/Ro60 antibodies, in contrast to idiopathic castleman disease

**DOI:** 10.1038/s41598-024-53413-5

**Published:** 2024-02-05

**Authors:** Mirei Shirakashi, Yuri Nishida, Ran Nakashima, Masakazu Fujimoto, Ryosuke Hiwa, Hideaki Tsuji, Koji Kitagori, Shuji Akizuki, Akio Morinobu, Hajime Yoshifuji

**Affiliations:** 1 Department of Rheumatology and Clinical Immunology, Graduate School of Medicine, Kyoto University, 54 Kawaharacho, Shogoin, Sakyo-ku, Kyoto, 606-8507 Japan; 2 Department of Diagnostic Pathology, Graduate School of Medicine, Kyoto University, Kyoto, Japan

**Keywords:** Autoimmunity, Connective tissue diseases, Lymph node

## Abstract

TAFRO syndrome is an acute systemic inflammatory disease characterized by thrombocytopenia, anasarca, fever, reticulin fibrosis/renal dysfunction, and organomegaly. There have been increasing reports that TAFRO is a disease distinct from idiopathic multicentric Castleman disease and that TAFRO patients may be positive for anti-SSA antibodies. To assess anti-SSA antibody positivity and the clinical characteristics of the two diseases, we retrospectively compared 7 TAFRO and 10 iMCD patients in our hospital. The mean age of onset of TAFRO and iMCD was 48.0 (interquartile range [IQR], 41–53) and 45.0 (IQR, 35–53) years, respectively. The TAFRO and iMCD groups had 6 (86%) and 4 (40%) male patients, respectively, and the following pretreatment laboratory values: platelet count, 3.8 (IQR, 2.2–6.4) and 35.5 (IQR, 22.2–42.8) × 10^4^/μL, respectively; C-reactive protein, 10.2 (IQR, 6.8–21.4) and 9.5 (IQR, 6.2–13.6) mg/dL, respectively; IgG, 1431 (IQR, 1112–1815) and 4725 (IQR, 3755–5121) mg/dL, respectively. RNA immunoprecipitation (5 cases for anti-SSA) or protein array (5 cases for anti-SSA/Ro60) detected anti-SSA antibodies in six (86%) TAFRO patients but not in iMCD patients; it did not detect anti-SSB antibodies in any of the patients. None of the patients were diagnosed with Sjögren syndrome. All iMCD patients treated with tocilizumab (TCZ) responded well. Meanwhile, two of six TAFRO patients treated with TCZ showed inadequate responses; thus, both patients were switched to rituximab, following which they achieved remission. TAFRO and iMCD have different clinical features. TAFRO may be categorized as a severe phenotype of the anti-SSA antibody syndrome.

## Introduction

TAFRO syndrome is a systemic inflammatory disease characterized by thrombocytopenia, anasarca, fever, reticulin myelofibrosis/renal dysfunction, and organomegaly with or without lymphatic tissue^1,2^. It is an extremely rare disease estimated to occur in one out of a million individuals in Japan, with an unknown cause, often having an acute onset and a tendency to become severe^2^. Several diagnostic criteria have been proposed for TAFRO^2,3^. Although TAFRO has been categorized as a subtype of idiopathic multicentric Castleman disease (iMCD), it has been interpreted as having different clinical features^4^. A study reported that TAFRO with characteristic lymph node findings consistent with iMCD is called iMCD-TAFRO and that TAFRO is associated with diseases other than iMCD^5^. However, some TAFRO patients have been diagnosed without a lymph node biopsy, and their clinical features did not differ from those of iMCD-TAFRO^6^. Further case–control studies are required to clarify the relationship between TAFRO and iMCD.

In recent years, there has been an increasing number of reports of positive anti-SSA antibodies in patients diagnosed with TAFRO; in a previous report, all Sjögren syndrome (SS) patients with TAFRO-like symptoms were positive for anti-SSA antibodies^7^, whereas in other studies, some patients with positive anti-SSA antibodies did not have SS^8,9^. Although iMCD complications with SS^10,11^ have been reported, they were diagnosed separately. Based on these findings, we hypothesized that TAFRO may be related to anti-SSA antibodies. This study aimed to determine the differences between TAFRO and iMCD. We compared the clinical characteristics, autoantibodies, and treatment responses of TAFRO patients with those of iMCD patients.

## Methods

### Patients

We retrospectively enrolled 7 TAFRO and 10 iMCD patients at Kyoto University Hospital from 2006 to 2021 in this study. TAFRO occurs in approximately one in a million individuals^2^, while iMCD is estimated to affect around 1500 people across Japan. In Kyoto, this would translate to roughly 2–3 cases of TAFRO and around 30 cases of iMCD. Considering the patient population that has been managed at our institution, the sample size in this study is reasonable. The diagnosis of TAFRO was based on the 2019 Updated Diagnostic Criteria and Disease Severity Classification for TAFRO Syndrome^2^ or the Proposed Diagnostic Criteria for TAFRO-iMCD^4^. The diagnosis of iMCD was based on international evidence-based consensus diagnostic criteria for human herpesvirus 8–negative/iMCD^12^. Sera from all patients were collected at the time of hospitalization and stored at − 30 °C.

### Ro60 and Ro52 autoantibody detection

Detection of SS-A antibodies was profiled using A-Cube (ProteoBridge, Tokyo, Japan), a multiplex wet protein array covering 33 target antigens of 28 autoantibodies that are associated with SSc^13^.

Ro60 antigen includes full-length RO60 (TROVE2), according to UniProt accession number P10155. Ro52 antigen includes TRIM21 spanning amino acid residues 1–400, according to UniProt accession number P19474.

The proteins synthesis with GST and FLAG tags added on their N-terminus was performed using the wheat germ cell-free translation system. The proteins were spotted onto GSH-coated glass plates using independent cylinder system (BIOTEC, Tokyo, Japan). Human serums were diluted by 3:1000, added to the protein arrays, and reacted for 1 h at room temperature. Next, the protein arrays were washed, and goat anti-Human IgG (H + L) Alexa Flour 647 conjugate (Thermo Fisher Scientific, San Jose, CA, USA) diluted 1000-fold was added to the protein arrays and reacted for 1 h at room temperature. Finally, the protein arrays were washed, air-dried, and fluorescent images were acquired using a fluorescence imager (GenePix 4000B, (Molecular Devices, San Jose, CA, USA). Fluorescence images were analyzed to quantify autoantibodies.

### Immunoprecipitation

The presence of anti-SSA and anti-SSB antibodies were determined via the RNA immunoprecipitation (IP) assay using HeLa cell extracts, as described previously^14^. Briefly, 10 μL of patient serum was mixed with 2 mg of protein A–Sepharose CL-4B (GE Healthcare, Chicago, IL, USA), following which 500 μL of IP buffer (10 mM Tris–HCl, pH 8.0, 500 mM NaCl, 0.1% Nonidet P-40) was added, and incubation was carried out on a rotator for 2 h at 4 °C. The IgG-coated Sepharose was washed four times in 500 μL of IP buffer using 10-s spins in a microfuge; then, it was resuspended in 400 μL of NET-2 buffer (50 mM Tris–HCl, pH 7.5, 150 mM NaCl, 0.05% Nonidet P-40). This suspension was incubated with 300 μL of HeLa cell extract, derived from 6 × 10^6^ cells, on the rotator for 2 h at 4 °C. The antigen-bound Sepharose beads were then collected via centrifugation for 10 s in the microfuge, washed four times with 500 μL of NET-2 buffer, and resuspended in 270 μL of NET-2 buffer. To extract bound RNAs, 30 μL of 3.0 M sodium acetate, 15 μL of 20% sodium dodecyl sulfate, and 300 μL of phenol/chloroform/isoamyl alcohol (50:50:1, containing 0.1% 8-hydroxyquinoline) were added to the Sepharose beads. After agitation in a vortex mixer and spinning for 1 min, RNAs were recovered in the aqueous phase after ethanol precipitation, following which they were dissolved in 20 μL of electrophoresis sample buffer composed of 10 M urea, 0.025% bromophenol blue, and 0.02% xylene cyanol-FF in TBE buffer (90 mM Tris–HCl, pH 8.6, 90 mM boric acid, and 1 mM EDTA). The RNA samples were denatured at 65 °C for 5 min and then resolved in 7 M urea–10% polyacrylamide gels, which were then silver-stained (Bio-Rad Laboratories, Hercules, CA, USA).

### Histological evaluation

Histopathological classification based on lymph nodes or bone marrow was determined by a pathologist (M.F.). The lymph node types were classified as hyaline vascular (HV), plasma cell (PC), mixed (HV + PC) type^15^. Bone marrow was determined by increased megakaryocyte count and fibrosis^16^.

### Statistical analyses

Statistical analyses were performed using Fisher’s exact test for comparison of frequencies, and Mann–Whitney U-test for comparison of continuous variables. The data were analyzed using JMP^®^ software (SAS Institute Inc., Cary, NC, USA). P < 0.05 was considered to indicate statistical significance.

### Ethical approval and consent participate

This study was approved by the Ethics Committee of Kyoto University Graduate School and Faculty of Medicine (Approval number: R1540) and was conducted in accordance with the Declaration of Helsinki and Health Insurance Portability and Accountability Act.

## Results

### Baseline clinical characteristics and laboratory data of TAFRO and iMCD patients

The pretreatment clinical and serological characteristics of the seven TAFRO and 10 iMCD patients were compared (Table 1). There were no differences in age or sex. Edema, pleural effusion, hepatosplenomegaly, thrombocytopenia, and elevated levels of lactate dehydrogenase, alkaline phosphatase, and creatinine were more frequent in TAFRO patients than in iMCD patients. iMCD patients showed higher IgG levels and greater lung involvement than TAFRO patients, whereas no differences were observed in hemoglobin level, albumin level, C-reactive protein level, or fever between the two groups.

**Table 1:** Baseline clinical characteristics and laboratory data of TAFRO and iMCD patients.

Clinical characteristics	TAFRO (n = 7)	iMCD (n = 10)	p value
Age in years, median (IQR)	48 [41–53]	45 [35–53]	0.69
Male, n (%)	6 (86%)	4 (40%)	0.13
Edema, n (%)	7 (100%)	2 (20%)	0.0023
Pleural effusion/Ascites, n (%)	7 (100%)	1 (10%)	0.0004
Fever (> 37.5 ℃), n (%)	6 (86%)	4 (40%)	0.13
Lymphadenopathy, n (%)	6 (86%)	10 (100%)	0.41
Hepatosplenomegaly, n (%)	6 (86%)	3 (30%)	0.0498
Lung lesions, n (%)	0 (0%)	6 (60%)	0.035
Laboratory data, median (IQR)			
PLT (× 10^4^/μL)	3.8 [2.2–6.4]	35.5 [22.2–42.8]	0.0004
Hb (mg/dL)	8.6 [7.8–9.0]	8.7 [8.4–9.0]	0.74
WBC (× 10^3^/μL)	11.9 [10.4–16.2]	10.9 [7.7–11.4]	0.10
Alb (g/dL)	2.0 [1.6–2.3]	2.5 [2.4–2.9]	0.10
LDH (U/L)	243 [230–294]	157 [123–208]	0.0095
ALP (U/L)	831 [460–1299]	280 [249–416]	0.025
Cre (mg/dL)	1.7 [1.5–2.3]	0.9 [0.6–1.1]	0.00051
CRP (mg/dL)	10.2 [6.8–21.4]	9.5 [6.2–13.6]	0.47
ESR (mm/h)	85 [57–89] (n = 6)	115 [95–117] (n = 9)	0.046
IgG (mg/dL)	1432 [1112–1815]	4725 [3755–5121]	0.0004
sIL-2R (U/mL)	918 [473–1250]	1210 [926–2240]	0.25

Data are expressed as median (IQR), or number (percentage). The difference of frequencies was tested by Fisher’s exact test and the continuous variables was tested by Mann–Whitney U test. P-values of 0.05 or less were considered statistically significant.

*PLT* platelet, *Hb* hemoglobin, *WBC* white blood cell, *Alb* albumin, *LDH* lactate dehydrogenase, *ALP* alkaline phosphatase, *Cre* creatinine, *CRP* c-reactive protein, ESR:erythrocyte sedimentation rate, *IgG* immunoglobulin G, *sIL-2R* soluble interleukin-2 receptor.

### Histological findings of TAFRO and iMCD patients

Next, we compared the pathological findings in the lymph nodes and bone marrow (Table 2). Lymph node biopsies were performed in 5 TAFRO and all iMCD patients and revealed HV, PC, and mixed types. Their proportions showed no significant differences between TAFRO and iMCD patients. Bone marrow biopsy was performed in five TAFRO patients and four iMCD patients. Bone marrow fibrosis and increased megakaryocyte count were observed only in the TAFRO group.

**Table 2:** Histological findings of TAFRO and iMCD.

Lymph nodes	TAFRO (n = 5)	iMCD (n = 10)	p value
Hyaline vascular (HV)	1 (20%)	0 (0%)	1.00
Plasma cell (PC)	2 (40%)	7 (70%)	0.33
Mixed (HV + PC)	2 (20%)	3 (30%)	1.00

Bone marrow	TAFRO (n = 5)	iMCD (n = 4)	p value
Reticulin myelofibrosis	3 (60%)	0 (0%)	0.17
Increased number of megakaryocytes	3 (43%)	0 (0%)	0.23

Data are expressed as number (percentage). The difference of frequencies was tested by Fisher’s exact. P-values of 0.05 or less were considered statistically significant. Mixed type had areas of both the hyaline vascular and plasma cell variant. Lymph nodes biopsies were performed in 5 cases with TAFRO. Bone marrow biopsies were performed in 5 cases with TAFRO and 4 cases with iMCD.

### Evaluation of anti-SSA antibody

Anti-SSA antibodies can be distinguished based on their antigenic target proteins, either anti-SSA/Ro60 and SSA/Ro52 antibodies. The presence of anti-SSA/Ro60 and SSA/Ro52 antibodies was determined using protein array (Table 3). In TAFRO anti-SSA/Ro60 antibodies were positive in 5 cases (71.4%) and anti-SSA/Ro52 antibodies were positive in 2 cases (28.6%) and inconclusive in 1 case (14.3%). In iMCD, neither anti-SSA/Ro60 and SSA/Ro52 antibodies were detected in any of the cases. Both of protein array and RNA-IP were detected in four cases (Patient (Pt) No 1, 2, 3, 5), Pt No 4 was detected only in RNA-IP and Pt No 6was detected only in the protein array. In other words, six patients (85.7%) in whom anti-SSA antibodies using protein array or RNA-IP appeared on TAFRO, while no cases positive for either anti-SSA, anti-SSA/Ro60 or anti-SSA/Ro52 were observed in iMCD.

**Table 3:** Clinical manifestations of TAFRO and iMCD patients.

Patient's number	TAFRO							iMCD									
	1	2	3	4	5	6	7	8	9	10	11	12	13	14	15	16	17
Age in years/Sex	47/M	29/M	48/M	42/F	60/M	54/M	48/M	68/F	25/M	43/F	62/M	55/M	36/F	15/F	48/M	47/F	35/F
Thrombocytopenia	+	+	+	−	+	+	+	−	−	−	+	−	−	−	−	−	−
Anasarca	+	+	+	+	+	+	+	+	−	−	+	−	−	−	−	−	−
Fever	+	+	+	+	+	−	+	+	+	−	−	+	−	−	+	−	−
Reticulin fibrosis	+	N/A	−	N/A	+	+	−	−	N/A	N/A	−	−	N/A	N/A	N/A	−	N/A
Organomegaly	+	+	+	+	+	+	+	+	+	+	+	+	+	+	+	+	+
Histology of lymph nodes	PC	PC	Mix	Mix	N.D.	HV	N.D.	PC	PC	PC	PC	Mix	Mix	PC	Mix	PC	PC
Anti-SS-A/Ro60 antibody (index value)	59.9	253.1	21.6	2.7	126	21	2.0	0.0	3.0	0.2	0.2	0.3	0.0	1.2	1.4	1.9	2.2
Anti-SS-A/Ro52 antibody (index value)	33.4	17.4	2.3	6.7	5.1	11.4	2.3	0.0	0.0	0.0	1.1	0.5	2.5	0.3	1.1	1.6	3.7
RNA-IPP	SS-A	SS-A	SS-A	SS-A	SS-A	−	−	−	−	−	−	−	−	−	−	−	−
Treatment																	
PSL	+	+	+	+	+	+	+	−	+	+	+	−	+	−	−	+	+
TCZ	+	+	+	+	+	−	+	+	+	+	+	+	−	+	+	+	+
Effective	RTX	TCZ	RTX	PSL, TCZ	PSL, TCZ, CyA	PSL, CyA	PSL, TCZ	TCZ	PSL, TCZ	PSL, TCZ	PSL, TCZ	TCZ	PSL	TCZ	TCZ	PSL, TCZ	PSL, TCZ

Anti-SS-A antibodies was detected using a multiplex protein array. Anti-SS-A/Ro60 antibody index values <7 were considered negative, 7-10 were inconclusive, and >10 were positive. Anti-SS-A/Ro52 antibody index values <10 were considered negative, 10-13 were inconclusive, and >13 were positive.

*N/A* not available (#5 and #7 were not performed lymph node biopsies. #2, #4, #9, #10, #13, #14, #15, #17 were not performed bone marrow biopsies), *N.D.* not detected, *PSL* prednisolone, *TCZ* tocilizumab, *RTX* rituximab, *CyA* cyclosporin A.

**Figure 1:**
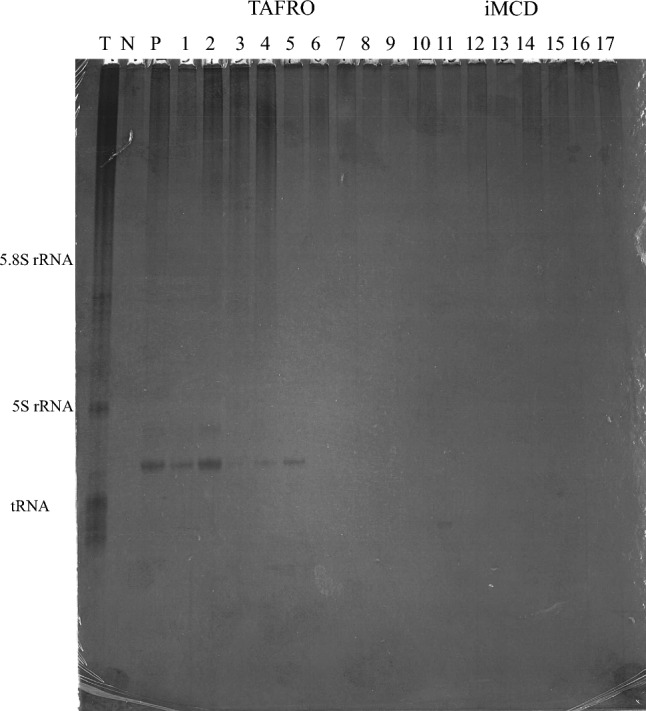
Immunoprecipitation of nucleic acids with sera patients with TAFRO and iMCD patients. *T* total nucleic acids, *N* negative control (normal healthy control), *P* anti-SSA antibody positive control (SS’s patient), Lanes 1–7: sera from TAFRO patients (patient’s number #1–7), Lanes 8–17: sera from iMCD patients (patient’s number #8–17).

Six patients with anti-SSA antibody-positive TAFRO were examined to determine whether they fulfilled the diagnosis of SS^17^ (Table 4). RNA-IP showed that none of the sera contained anti-SSB antibodies (Fig. 1). None of the patients had subjective symptoms of dryness; however, objective tests were performed after obtaining consent. Saxon and unstimulated whole sialometry tests yielded positive results in three (50%) of six patients, and Schirmer test yielded a positive result in none of five patients who consented to the measurement; meanwhile, none of the five patients who consented to fluorescent dye test showed a positive result. The possibility of a diagnosis of SS in all cased remains, and a lip biopsy was not performed in any case, due to low platelet count, severe systemic condition, and inability to obtain consent.

**Table 4:** Determination of SS diagnostic criteria.

Patient's number	1	2	3	4	5	6
Anti-SSA antibody	+	+	+	+	+	+
Anti-SSB antibody	−	−	−	−	−	−
Saxon test or unstimulated whole sialemetery	−	+	+	+	−	−
Schirmer test	−	−	+	−	−	N/A
Ocular surface staining	−	−	−	−	−	N/A

The presence of anti-SSA antibody and anti-SSB antibody were determined by Protein array or RNA-IP.

*N/A* not available.

### Efficacies of the treatments

Finally, the efficacies of these treatments were examined. iMCD was treated with prednisolone (PSL) alone in one case, tocilizumab (TCZ) alone in five cases, and a combination of PSL and TCZ in four cases, and all treatments were effective. In the TAFRO group, no cases were treated with PSL or TCZ alone, two cases were treated with the PSL and TCZ combination, one case was treated with PSL and cyclosporine A (CyA), and one case was treated with the concomitant use of PSL, CyA, and TCZ. The PSL and/or TCZ treatment was effective in five TAFRO cases (71.4%) but ineffective in the remaining two cases (28.6%). Remission was achieved after switching to rituximab in the two refractory cases; these two patients tested positive for anti-SSA antibodies.

## Discussion

**Figure 2:**
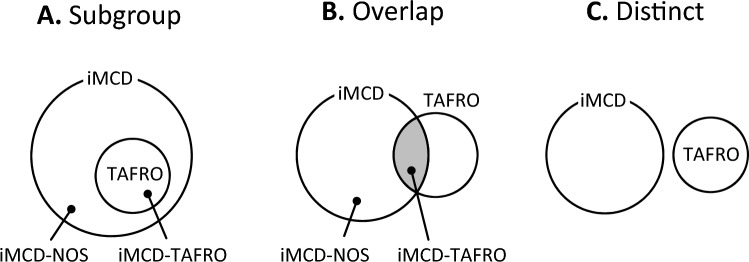
Disease concept of iMCD and TAFRO. *NOS* Not otherwise specified.

TAFRO is a relatively recently proposed disease, and its definition is still undergoing revision. There are two main diagnostic criteria for TAFRO^2,4^. Lymph node pathology is not required for the Masaki et al. criteria, but both criteria require the exclusion of autoimmune diseases or infections. TAFRO is believed to be a subgroup of iMCD (Fig. 2A), but there is a school of thought that TAFRO and iMCD are independent groups (Fig. 2B,C). Because of the difference in disease concepts, the requirement of pathological findings may differ between the two diagnostic criteria. Invasive tests are difficult to perform when the disease is unstable. In such a situation, an interpretation was reported to separate iMCD-TAFRO, which meets lymph node pathology criteria, from TAFRO, which is related to the exclusion diagnosis^5^. Thus, the diagnostic criteria for TAFRO have become more complex and require an obvious concept in practice. In this study, two of the TAFRO cases had no lymphadenopathy on the body surface, making biopsy difficult given their general condition. These two cases were diagnosed with TAFRO using the diagnostic criteria of Masaki et al.^5^.

In recent years, there have been many reports on the relationship between TAFRO and anti-SSA antibodies, and some patients have been diagnosed with both SS and TAFRO^7,17–23^. In addition, in some cases, patients tested positive for anti-SSA antibodies but did not undergo lip biopsy or oral or ophthalmological examination because of the absence of symptoms of dryness^24–29^. Some of them were also positive for anti-SSB antibodies, which are highly specific for SS^8,29^. In cases with a confirmed diagnosis of SS, TAFRO and SS were diagnosed simultaneously, or SS preceded TAFRO^30^.

These findings suggest two possible explanations for TAFRO. First, TAFRO is a severe form of SS. Some cases of SS with protein-losing enteropathy have pleural ascites and generalized edema^31^; renal dysfunction and thrombocytopenia are observed in 4% and 8.1% of SS patients, respectively^32^. Moreover, myelofibrosis has been reported in a few cases^33^. In the present cases, the patients did not have symptoms of dryness, but the objective assessment of dryness was positive in three of six patients. Since lip biopsy and salivary gland scintigraphy were refused in all cases because the patients were not troubled by the subjective symptoms, but the possibility of a diagnosis of SS remains; under the ACR/EULAR diagnostic criteria^34^, three patients would have been diagnosed with SS if only objective measures were used, but since subjective symptoms of dryness were required, a diagnosis of SS could not be made.

The second possible explanation is that TAFRO is an autoimmune disease characterized by the presence of anti-SSA antibodies. During 4.5 and 8 years of follow-up, 12.2% and 19% of undifferentiated connective tissue disease cases positive for anti-SSA antibodies were diagnosed with SS, respectively^35,36^. This suggests that antibody-positive cases may develop into SS in the future.

SS-A/Ro is part of the Ro/La heteroantigen complex, which consists of three unique proteins (52 kDa Ro, 60 kDa Ro and La) and four small RNAs particles^37^. Recently, there has been an increasing number of studies elucidating the background of anti-SSA/Ro60 and SSA/Ro52 antibodies within the SS-A antibody group. It has been reported that in cases where anti-SSA/Ro52 antibodies are positive alone, the proportion of non-autoimmune diseases is approximately 40%, which is higher compared to cases where anti-SSA/Ro60 antibodies are positive alone or both are positive^38^. In this study, there were no individuals with anti-SSA/Ro52 positive alone, but there were 3 cases with anti-SSA/Ro60 positive alone and 2 cases with positivity for both antibodies (one case was positive in RNA-IP but negative in the protein array, so it is unclear which one is being recognized). This supports the possibility that TAFRO may exhibit characteristics of autoimmune diseases.

In addition, patients with anti-SSA/Ro60 antibodies exhibit elevated expression of interferon type 1 (IFN-I)–related genes, regardless of the disease^39^. The mTOR pathway is activated in TAFRO, and the expression of IFN-I–related genes increase^40^. IFN-I is known to induce thrombotic microangiopathy (TMA)^41^. In TAFRO, there have been reports of manifestations such as thrombocytopenia and renal impairment, with renal biopsies revealing TMA-like glomerulopathy^42^. In severe cases of COVID-19, the level of IFN-I has been increased over time^43^. A Case that presented with TAFRO-like symptoms after the onset of COVID-19 was positive for anti-SS-A antibody^44^. Based on the above, it is possible that the IFN-I response　triggered by such as viral infection may be involved in the development of both anti-SS-A antibodies and TAFRO-like manifestations.

**Figure 3:**
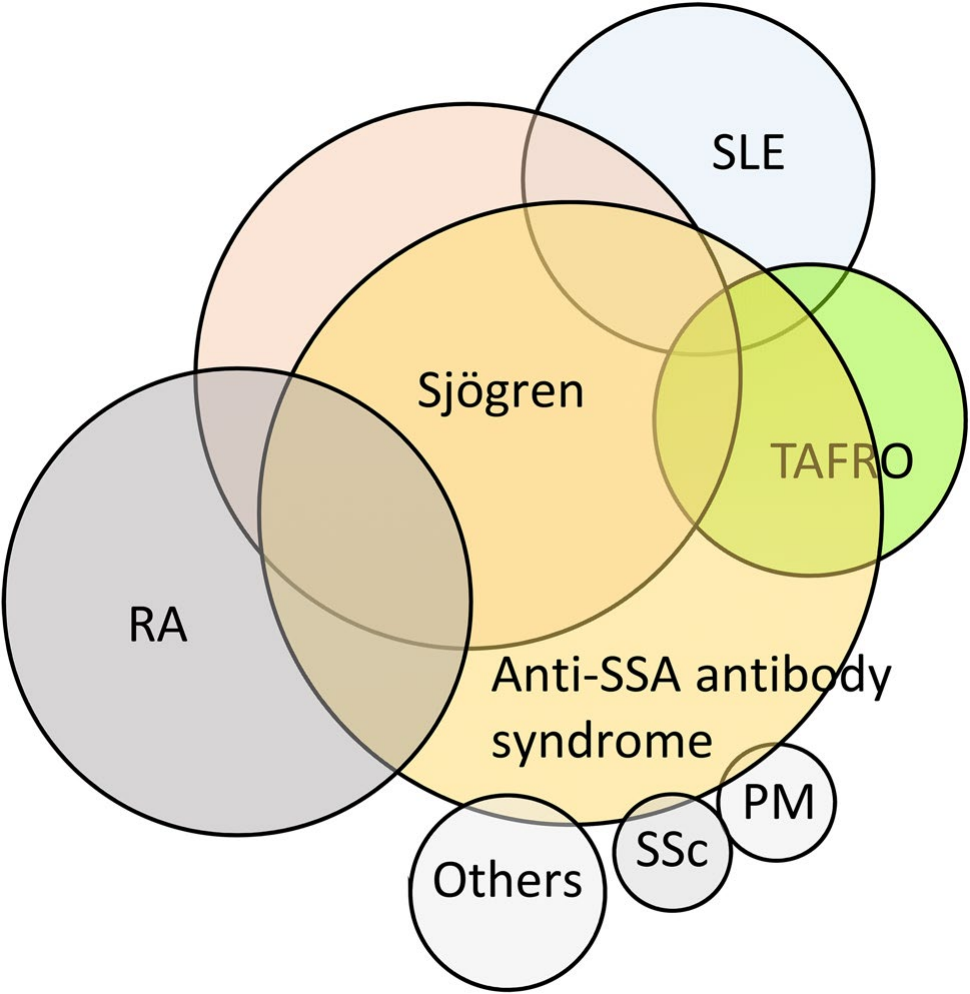
Hypothesis of concepts of TAFRO and anti-SSA antibody syndrome. *SLE* systemic lupus erythematosus, *RA* rheumatoid arthritis, *SSc* systemic sclerosis, *PM* polymyositis, *DM* dermatomyositis.

Anti-SSA/Ro60 and SSA/Ro52 antibodies have different prevalence in autoimmune diseases. Patients with SS most commonly tested positive for both antibodies^38^. However, in systemic lupus erythematosus (SLE), the prevalence of anti-SSA/Ro60-positive alone and both antibodies-positive was similar^38^. On the other hand, in systemic sclerosis (SSc) and polymyositis/dermatomyositis (PM/DM), there was a tendency for anti-Ro52 antibody-positive alone to be more common^38^. In cases of TAFRO, both anti-Ro60 antibody-positive alone and both antibodies-positive were observed. In a report on TAFRO cases that met the SLE criteria, only half of the patients tested positive for anti-dsDNA antibodies (there was no mention of anti-SSA antibodies) and all patients were older men, which is not typical of SLE^45^. Furthermore, anti-SSA/Ro60 antibody-positive SS patients tended to lack characteristic glandular lesions and not exhibit typical features^46^. Based on these findings, even when TAFRO patients meet the criteria for SLE or SS, they may not be typical cases but rather belong to a group of disorders characterized by the presence of anti-SSA/Ro60 antibodies. Diseases with anti-SSA antibodies are summarized in Fig. 3.

TAFRO often has an acute onset and severe presentation, with some cases taking a fatal course. Treatment often takes precedence over diagnosis in clinical practice. The present study and literature review suggest that some cases of TAFRO were associated with anti-SSA antibodies. We believe that that considering the pathophysiology associated with anti-SSA antibodies even in the absence of subjective symptoms of sicca will contribute to a new concept of TAFRO. Additionally, lymph node biopsy is effective in excluding tumors, such as malignant lymphoma, and should be performed in consideration of the patient's condition if it cannot be clearly excluded^2^.

The limitations of this study include the small sample size and the lack of clarity regarding the diagnostic criteria for SS. Due to the rarity of TAFRO onset, it was extremely challenging to gather cases from a single facility, making sample collection highly difficult. Additionally, since lip biopsy was not performed, the evaluation of SS diagnosis could not be conducted thoroughly. Consent could not be obtained from the patients, and given the severity of each case and low platelet counts, ensuring the safety of conducting biopsies was unattainable, leading to the decision to abandon this aspect. There may be a bias in the increased proportion of patients with autoimmune conditions because our section focused on rheumatology, rather than hematology. In the future, it is crucial to collect a larger number of cases from multiple facilities for equivalent validation, and long-term observation is necessary to determine whether patients with anti-SSA antibodies will meet the definitive criteria for SS.

## Conclusions

TAFRO has a high rate of anti-SSA antibody positivity along with clinical characteristics different from those previously reported in cases of iMCD, suggesting that it may be a distinct disorder from iMCD. TAFRO syndrome may be associated with anti-SSA antibody and when TAFRO or iMCD is suspected, it is important to screen for autoantibodies.

## Data Availability

The underlying data presented in this study is available upon reasonable request to the corresponding author.
